# Physico-Chemical, Microbiological and Sensory Properties of Water Kefir Drinks Produced from Demineralized Whey and Dimrit and Shiraz Grape Varieties

**DOI:** 10.3390/foods12091851

**Published:** 2023-04-29

**Authors:** Havva Şafak, İlhan Gün, Milna Tudor Kalit, Samir Kalit

**Affiliations:** 1 Health Sciences Institute, Department of Animal Products Hygiene and Technology, Burdur Mehmet Akif Ersoy University, 15100 Burdur, Türkiye; 2 Food Processing Department, Burdur Vocational School of Food, Agriculture and Livestock, Burdur Mehmet Akif Ersoy University, 15100 Burdur, Türkiye; 3 Department of Dairy Science, University of Zagreb Faculty of Agriculture, Svetošimunska cesta 25, 10000 Zagreb, Croatia

**Keywords:** water kefir, demineralized whey, grape juice, raisins, Dimrit grape, Shiraz grape

## Abstract

Water kefir grains cannot grow in milk. Therefore, the aim of this study was to investigate whether water kefir grains can show activity in demineralized whey, an environment containing lactose as a carbon source. The physicochemical, microbiological and sensory properties of water kefir prepared from demineralized whey containing 2% and 5% lactose and raisins or grape juice from two grape varieties (Dimrit and Shiraz) were investigated. It was found that the protein content of the water kefir increased significantly (*p* < 0.05), especially when grape juice was added. The total soluble solids and viscosity of the samples with grape addition increased significantly (*p* < 0.05). Total phenolic content and antioxidant capacity increased significantly with grape addition (*p* < 0.05), with the effect of Shiraz grape being more pronounced. In general, it was found that the content of K, P, Na, Ca and Mg was higher in the samples with grape addition. The sensory properties of water kefir made from dWhey with 2% lactose and grape juice were better. It was also confirmed that viability of water kefir microbiota is better in water kefir drink made from dWhey with 2% lactose due to higher pH value in comparison to dWhey with 5% lactose.

## 1. Introduction

The demand for functional products is rapidly increasing worldwide, and in recent years consumers prefer alternative probiotic drinks such as water kefir or kombucha [1,2]. Water kefir is produced via fermentation of carbohydrate-containing solution by water kefir grains, which contain many probiotic microorganisms. Various carbohydrate sources such as sucrose, honey and fresh or dried fruits can be used to produce water kefir [3]. Depending on the microbiota of the water kefir grains and the carbohydrate source used in its production, this beverage includes many species such as: lactic acid bacteria *Lactobacillus brevis*, *L. kefir*, *L. acidofilus*, *L. casei*, *L. caucasicus* and *L. bulgaricus*; leuconostocci *Leuconostoc dextranicum*; yeasts *Acetobacter aceti*, *A. rasens*, *Kluvyveromyces marxianus*, *Torulaspora delbrueckii*, *Torula kefir*, *Candida kefir* and *Saccharomyces cerevisia*; and streptococci *Streptococcus lactis*, *Str. durans*, *Str. cremoris*, *Str. citrovorum*, and *Str. lactis subsp diacetylactis* [4,5,6].

In cheese production, about 90% of the milk is discarded as whey [7]. It is considered the biggest waste of the dairy sector, but due to its numerous health benefits such as immune system improvement, antimicrobial, antiviral and antifungal properties, whey can be used for the development of functional foods [8] such as albumin cheese, lactose, whey powder, whey protein powder and drinks with probiotic bacteria [9,10,11,12,13,14]. In the production of dried whey products, the whey can be dried directly or its components can be separated to obtain different products such as lactose-free whey powder, whey protein isolate, whey protein concentrate and demineralized whey (dWhey) [15,16]. 

Yamahata [17] highlighted that dWhey, which contains 80% lactose, 13% protein, 1% fat, 1% ash and 5% moisture, is not suitable for maintaining the viability of microorganisms in kefir grains due to excessive lactose content. The ability of water kefir grains to ferment sucrose allows their use in making water kefir drinks from a variety of sugar sources, as mentioned above [18]. However, the use of milk sugar (lactose) as a carbon source in water kefir drinks has not yet been reported in the literature. One of the hypotheses of this study was to investigate whether water kefir grains, which cannot grow in milk, can show activity in an environment containing lactose but no minerals as a carbon source, and to determine the quality of the product obtained. The substrate composition is a very important parameter for the fermentation of water kefir. The appropriate choice of carbon and nitrogen source in water kefir drink production affects the growth of water kefir grains, on the one hand, and the fermentation capacity of the microbiota of the grains, on the other hand. Sugar is the main carbon source and fresh or dried fruits are used as nitrogen source. However, it is known that the calcium content of the water used to produce the sugar solution also plays a role [19]. Several studies have demonstrated a positive effect of the use of dried figs on the development of water kefir grains [20]. The combined effect of lactose and fruit sugar derivatives on the viability of the microbiota of water kefir grains and product quality has been investigated. Therefore, the aim of this study was to investigate the physicochemical, microbiological and sensory properties of water kefir drinks produced from demineralized whey containing 2% and 5% lactose and raisins or grape juice from two grape varieties (Dimrit and Shiraz).

## 2. Materials and Methods

Two grape varieties were used in this research. The Dimrit grape was obtained from Burdur province, while the Shiraz grape was obtained from the villages of Uşak province. Demineralized whey (dWhey) was obtained from Süt Ofis Gıda A.Ş. (Burdur, Türkiye), which produces whey powder in Burdur province.

### 2.1. Preparing of Grape Juice and Raisins

Freshly harvested grapes were brought to the laboratory, separated from the stems and washed. After the grapes were squeezed, the juice obtained was used in the production of water kefir beverages. The grape juice was pasteurized at 85 ± 1 °C for 5 min, then cooled to 15 ± 1 °C and centrifuged at 3000 rpm for 20 min. 

The traditional method from the Burdur region for drying grapes was used to produce raisins. For this purpose, ash from wood burning was added to the water. This mixture was boiled for 15 min, cooled and the aqueous phase was filtered. Olive oil (20 mL/L water) was added to the filtered water and the grapes were treated with this water before drying. The drying of the grapes was carried out over 4 days. 

### 2.2. Preparing of Water Kefir Drinks

Carbohydrate-based solutions containing 2% and 5% lactose were used to produce water kefir drinks (control groups) due to the fact that optimal carbohydrate content for water kefir grain microbiota growth is between 2% and 10% [21]. Two solutions with different lactose content were prepared using dWhey liquid concentrate containing 80% lactose, mixed with previously boiled water for 15 min and cooled to room temperature. The lactose-adjusted solutions (2% and 5%) were cooled to 25 °C and 3% water kefir grains (100% organic, live starter culture; Wellness–Drinks, Frankfurt am Mein, Germany) were added to each of the solutions. The first fermentation lasted 48 h. The fermented liquids were then filtered through sterile plastic sieves. After filtration, each water kefir with different lactose content was divided into five portions. To each water kefir with different lactose content, previously prepared raisins or grape juice (10%) from two grape varieties (Dimrit and Shiraz) was added according to Table 1.

**Figure 1:**
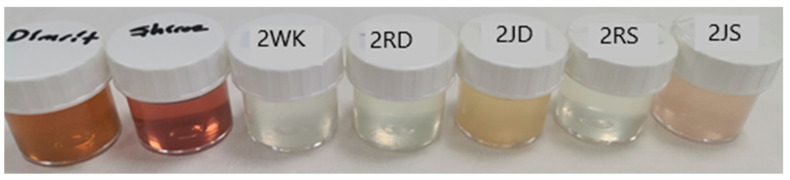
Samples of grape juices and water kefir drinks produced from demineralized whey (dWhey) with 2% lactose content. 2WK = 2% lactose; 2RD = 2% lactose + raisin of Dimrit; 2JD = 2% lactose + grape juice of Dimrit; 2RS = 2% lactose + raisin of Shiraz; 2JS = 2% lactose + grape juice of Shiraz.

**Figure 2:**
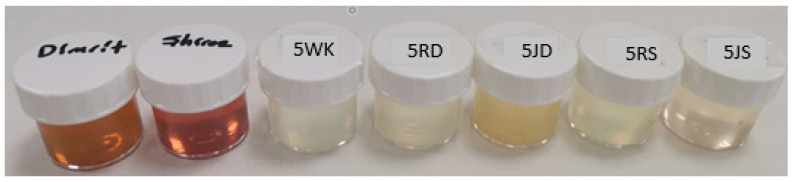
Samples of grape juices and water kefir drinks produced from demineralized whey (dWhey) with 5% lactose content. 5WK = 5% lactose; 5RD = 5% lactose + raisin of Dimrit; 5JD = 5% lactose + grape juice of Dimrit; 5RS = 5% lactose + raisin of Shiraz; 5JS = 5% lactose + grape juice of Shiraz.

The water kefir with the added grape varieties was then subjected to a second fermentation for 12 h. At the end of fermentation raisins were taken out, and the samples were analyzed (Figure 1 and Figure 2). Three batches of water kefir drinks were produced.

### 2.3. Physico-Chemical Analyzes

The pH values of the samples were measured using a Metler Toledo pH meter (SevenCompact, Switzerland). Total solids and ash content were analyzed using the gravimetric method [22]. Protein content was determined by the Kjeldahl method (ISO 8968-2). Total soluble solids content was determined as Brix value using an Atago Pal-1 Digital Refractometer (Japan). The viscosity values of the samples were determined using a spindle DV-2 at 100 rpm (Brookfield Viscosemeter, DV2T) [23]. The water activity values of the samples were determined using a portable hygrometer (Novasina AG, CH 8853, Labswift aw, Lachen, Switzerland). Sugar derivatives were determined by high performance liquid chromatography using a refractive index detector. Operating conditions for HPLC were: Mobile phase methanol:water (80:20), column temperature—30 °C and flow rate—1.0 mL/min [24]. The total phenolic content of the extracted grape samples was determined as gallic acid equivalent (mg/kg) according to the Folin–Ciocalteu method reported by [25]. Antioxidant activity was determined spectrophotometrically using the 2,2, -diphenyl-2-picryl-hydrazyl (DPPH) method [26]. Trolox equivalent antioxidant capacity (TEAC) antioxidant activity was determined according to Miller et al. [27]. In the analysis of volatiles, standard solutions (0.1 μL 2 methyl 3- heptanone and 6 μL 2 methyl-valeric acid in 1 mL internal standard) and 1 g NaCl were added to 5 g of sample and the samples were kept at 40 °C without fibers/20 min and 40 °C with fibers/20 min, then transferred to GC-MS with fiber and the peaks obtained from the samples were calculated using the software program. After 5 min at 40 °C, the GC-MS column temperature was increased to 230 °C (10 °C/1 min) and the total processing time was 90 min. Helium was used as the carrier gas and the flow rate was 1.2 mL/min [28]. Mineral content analysis was performed by ICP OES (inductively coupled plasma optical emission spectrometry, Australia) using a Perkin Elmer OPTIMA 5300 DV (Waltham, MA, USA) according to the EPA 6010 method and the values of Zn, P, Mn, Fe, Mg, Ca, Cu, Na and K were determined. The color analysis of the water kefir beverages was measured as CIE L*, a*, b* using a Minolta CR-400 colorimeter (Minolta Corp, Ramsey, NJ, USA). As the product was in liquid form, the color analysis was carried out in a special cylindrical device. All analyses were carried out in parallel for each batch.

### 2.4. Microbiological Analyzes

After adding 1 mL of water kefir to 9 mL of peptone water solution, serial dilutions were prepared under aseptic conditions. All microbiological analyses were performed using the pour plate method. The total number of mesophilic bacteria, the number of colonies growing on MRS agar, the number of colonies growing on M17 agar and the number of yeasts were analyzed. For the total number of mesophilic bacteria, 1 mL of sample was placed in Petri dishes, PCA (plate count agar) cooled to 45 °C was added to the Petri dishes and after 48 h of incubation at 30 °C, colonies were counted in Petri dishes containing 30–300 colonies. For colony counts growing on MRS agar, 15 mL of MRS agar (Merck) was added to samples cooled to 45 °C and after incubation at 37 °C for three days, colonies were counted in Petri dishes containing 30–300 colonies. For colony counting on M17 agar, samples were added to 15 mL M17 agar (Merck) Petri dishes and incubated at 37 °C for 2 days; counts were made in Petri dishes containing 30–300 colonies. For yeast counts, 1 mL of the sample was placed in Petri dishes, 15 mL of PDA (potato dextrose agar) cooled to 45 °C was poured into the Petri dishes and after incubation at 25 °C for 48–72 h, colonies were counted in Petri dishes containing 30–300 colonies [29].

### 2.5. Sensory Evaluation

The sensory evaluation was conducted according to the methods described by Lawless and Heymann [30] with some modifications. The sensory panel consisted of 10 panelists (aged 22–54 years, 4 females and 6 males) experienced in sensory evaluation of kefir drinks. The panelists were informed regarding the aim, protocols and methodology of the study and gave their written consent to participate. The samples were served in a glass cup. A rating scale was used for homogeneous and clear appearance (0–10), color (0–10), consistency (0–5), slightly sour smell (0–10), slightly yeasty taste (0–5), refreshing taste (0–5), pleasant fermented taste (0–5) and pleasant fruity taste (0–5).

### 2.6. Statistical Analysis

The results of the study were analyzed by repeated measures and one-way analysis of variance using the statistical program Minitab (Minitab Inc., State College, PA, USA, Version 16.1.0.). As a result of the analysis of variance, pairwise comparisons of the statistically significant characteristics were performed using the Tukey multiple comparison test. Significance was indicated by *p* < 0.05.

## 3. Results and Discussion

### 3.1. Physico-Chemical Composition

Table 2 shows the results of physico-chemical analyses of water kefir drinks. In general, all samples developed a pronounced acidity due to the fermentation of the water kefir by yeast as well as acetic acid bacteria groups and lactic acid bacteria. Silva et al. [31] reported a decrease in pH (from 6 to 4) of water kefir produced by fermentation with brown sugar for 48 h. The change in product pH significantly affects both product quality and probiotic bacteria viability. Therefore, in functional alcoholic beverages such as water kefir, the addition of sugar, fruit or vegetables and the duration of fermentation can result in significant changes [32]. In this study, significantly (*p* < 0.05), the highest pH was found in the control sample with 2% lactose (2WK), while the pH of water kefir with 5% lactose (5WK) was similar to that of 2WK (3.71 and 3.68, respectively). The addition of grapes significantly (*p* < 0.05) decreased the pH in water kefir drinks (Table 2). Thus, the pH of the water kefir drinks obtained by addition of Shiraz grape juice (2JS and 5JS) was significantly (*p* < 0.05) lower than the other samples analyzed. The results obtained in this study are within the range of those obtained by previous authors. Randazzo et al. [2] reported pH values of water kefir drinks with the addition of different fruits: apple 4.04, grape 3.8, kiwi 3.48, pomegranate 3.89, prickly pear 4.11 and quince 3.62. Çevik et al. [3] prepared water kefir drinks with different sugar derivatives and results show the highest pH of water kefir produced with refined sugar (5.62), but lower with honey (3.56) and grape pulp (4.06).

It was found that the total solids content of 2WK was similar to that of 2RS, while the other water kefirs with 2% lactose had significantly (*p* < 0.05) higher total solids content. The significantly highest (*p* < 0.05) total solids content was found in water kefir with 2% lactose and juice of traditional Dimrit grapes (2JD). The total solids content of the control sample of water kefir with a concentration of 5% lactose (5KW) was significantly (*p* < 0.05) the lowest compared to the other samples of the same lactose concentration. Statistical differences in total solids content were found in the samples containing raisins and grape juice (2RD vs. 2JS; 2RS vs. 2JS; 5RS vs. 5JS). Grape variety also had significant (*p* < 0.05) effect on total solids content (2RD vs. 2RS, 2JD vs. 2JS), which can be attributed to the different overall composition of the grapes. Dwiloka et al. [33] reported lower total solids content (3.8%) of water kefir with green coconut compared to the results of the present study. It is considered that the total dry matter content in water kefir drinks may be affected by the type and amount of fresh/dry fruit used as carbon source. When the lactose concentration in dWhey was increased from 2% to 5% and raisin and grape juice were added, the protein content increased significantly (*p* < 0.05) (Table 2). The protein from dWhey, raisins and grape juice thus influenced the nutritional value of the water kefir, albeit only slightly. Polat [34] found that different grape varieties such as Boğazdere, Öküzgözü and Şire have protein contents of 0.6%, 0.4% and 0.3%, respectively. Pocock et al. [35] studied the importance of harvesting technique in grape cultivation. They found that the protein content of grape juice of the Pinot Noir variety harvested by hand was 51 g/L, while it was 63 g/L for grape juice obtained by mechanical harvesting. For another grape variety, Sauvignon Blanc, the protein content of the grape juice was 203 g/L after hand harvesting and 205 g/L after machine harvesting. The protein content of the water kefir drink made from coconut was found to be 4.05–6.04%. Another study reported that the protein content decreased during fermentation, but the antioxidant activity increased due to the formation of bioactive peptides [33]. The reason for the low protein content in water kefirs produced using dWhey in our study is due to both the water used to reduce the lactose content and the filtration process used in dWhey production.

The ash content of the samples increased with increasing lactose content as well as with the use of raisins and grape juice, and this difference was statistically significant (*p* < 0.05) (Table 2).

It was found that the total soluble solids amount of the control sample (2WK) was significantly (*p* < 0.05) lower compared to other samples with the same lactose content. A statistical difference (*p* < 0.05) was also observed between the samples with raisins and grape juice and between the samples with different grape varieties (Table 2). The control sample with 5% lactose (5WK) had a significantly (*p* < 0.05) higher total soluble solids amount than the control sample with 2% lactose (2WK). In general, it was found that as the lactose concentration of the dWhey increased, the total soluble solids amount in the samples also increased. The total soluble solids amount in the production of water kefir varies depending on the lactose concentration and grape variety, which is due to the utilization of carbon sources in the environment during fermentation that convert into different metabolites, resulting in a decrease in the total soluble solids amount [5,36]. Gökırmaklı [37] stated that the total soluble solids amount during 48 h of fermentation decreased from 4.83% to 4.40% in the control sample and from 4.32% to 2.42% in the water kefir with figs.

The viscosity of the water kefir drinks varied depending on the lactose content and the addition of raisins or grape juice. The values were higher due to the increase in lactose concentration and showed significant (*p* < 0.05) differences between the samples to which raisins or grape juice were added. Significantly (*p* < 0.05), the highest viscosity values were found when grape juice (2DJ and 5JD) of the traditional Dimrit variety was used. Şen and Karaguel Yueceer [38] found a lower viscosity value (1.85 cP) for the whey drinks compared to the results of this study, which can be explained by the using demineralized whey and raisins or grape juice.

The water activity values (a_w_) of the samples varied depending on the carbohydrate content. Statistically significant (*p* < 0.05) lower a_w_ values were found in samples with 5% lactose compared to those with 2% lactose. Non-electrolytic solvents such as sugars and polyols cause a decrease in water activity due to their relationship to the number of hydroxyl groups in the molecule [39]. On the other hand, it was found that the effect of the addition of raisins and grape juice, as well as the grape variety, were insignificant with respect to the water activity of the produced water kefir (Table 2).

The total phenolic content of the water kefir drinks prepared with dWhey was significantly affected by both the lactose content, form of used grape (raisins or juice) as well as the grape variety (*p* < 0.05). In particular, the total phenolic content of the samples with added raisins or grape juice was significantly higher (*p* < 0.05) compared to the control samples. The total phenolic content in samples with added Shiraz grape juice was significantly (*p* < 0.05) higher in both cases (with 2% or 5% lactose) compared to the other samples (Table 2). The antioxidant DPPH activity of the samples obtained from Dimrit and Shiraz raisins and grape juices was significantly (*p* < 0.05) higher than that of the control samples (2WK and 5WK), which is consistent with the total phenolic content. Higher total phenolic content as a result of using raisins and grape juice in the production of water kefir significantly (*p* < 0.05) increased the inhibition rate of DPPH free radicals’ scavenging activity. When evaluated by grape variety, the samples obtained from Shiraz grape showed significantly (*p* < 0.05) higher DPPH antioxidant activity compared to the Dimrit grape, which is due to higher total phenolic content. The TEAC values of the samples containing dWhey with 5% lactose were also statistically (*p* < 0.05) higher than those of the samples containing 2% lactose, except for 2JS vs. 5JS. Similar to the DPPH value, the TEAC value of the water kefir drinks made with raisins was found to be significantly lower (*p* < 0.05) than the samples made with grape juice. When assessed by grape variety, it was found that the antioxidant TEAC capacity of products made with Shiraz grapes was significantly (*p* < 0.05) higher. Although grapes are among the fruits with the highest antioxidant capacity, the influence of the grape variety and the region where it is grown on the antioxidant properties is very important. Antioxidant capacity, which is proportional to flavonoid content in fruits and vegetables, is very important for health [40]. The DPPH radical scavenging activity of cherry, pomegranate and grape juice fermented with water kefir grains is 1456 mg/100 g, 42.08 mg/100 g and 588 mg/100 g, respectively, while the antioxidant TEAC values of cherry juice water kefir were found to be 7555 mg/100 g, grape juice water kefir 632 mg/100 g and pomegranate juice water kefir with no antioxidant value [41]. Although some lactic acid bacteria have the ability to scavenge reactive oxygen [42], the stability of antioxidant substances in foods can change with the pH of the product. For products that undergo a specific fermentation phase, such as water kefir, the change in pH has a significant impact on antioxidant capacity. In addition, water kefir fermentation can improve the antioxidant activity of the product through hydrolysis by microbial enzymes which results in the breakdown of the substrate into low molecules and the appearance of various bioactive compounds [43].

Both the lactose content and the grape variety and the form of grapes added influenced the mineral content of the water kefir drinks. An increase in lactose content in dWhey water kefir resulted in a significant increase (*p* < 0.05) in the content of K, Na, Ca, Mg and P, except for 2RD and 2RS water kefir drinks. A significant difference (*p* < 0.05) was observed for the content of minerals, except Cu, between dWhey water kefir beverages prepared with the addition of dried grapes and grape juice. The addition of raisins of both grape varieties to 2% lactose dWhey resulted in significantly (*p* < 0.05) higher levels of K, Na, Mg, Fe, Mn and P in the water kefir drinks, which was not the case for the samples containing 5% lactose (Table 3). For example, the Fe content of the water kefir drink made with Dimrit grape raisins (2RD) and 5RD) was about four times higher in the samples with raisins than in the samples with grape juice (2JD and 5JD). The most abundant mineral was K, and it was highest in the 2% lactose water kefir drinks with added raisins, 2RD (450.34 µg/g) and 2RS (333.87 µg/g). The content of K, Na, Mg, P, Fe and Zn was significantly higher (*p* < 0.05) in water kefir drinks with Dimrit grape raisins/juice compared to water kefir drinks made from Shiraz grapes. The samples produced with the Shiraz grape were found to all have significantly (*p* < 0.05) higher Ca content compared to the Dimrit grape. The mineral content of the water kefir drinks varied considerably, probably due not only to the grape, but also to the water used, as well as to the sequence of minerals passed during filtration. The soil structure of the region where the grapevine is grown and the care given to the grapevine can alter the mineral content of the grapes [44]. In a study by Meler [45], the nitrogen content of Shiraz grapes was determined to be 0.15–0.20%, phosphorus 0.02–0.03%, potassium 0.13–0.27%, calcium 0.01–0.04%, magnesium 0.01–0.16%, iron 13.8 mg/kg, manganese 4.30 mg/kg, zinc 2.49 mg/kg and copper 3.30 mg/kg. Minerals in the composition of water kefir beverages not only have a nutritional value, but are also important for the diversity of the microorganism group in the water kefir beverage [20].

dWhey has a greenish-yellow color. However, as it was diluted to adjust the lactose ratio during water kefir production, a slightly greenish-yellow color was observed (Table 4). Fermentation with water kefir grains significantly affected the color of the product and it took on a cloudy white color. The reason for the cloudy color is the breakdown of lactose during fermentation. In general, as the lactose content in dWhey increased, the L*, a* and b* values changed significantly (*p* < 0.05). It was observed that the color of dWhey-based water kefir beverages prepared by adding Dimrit grapes and Shiraz grapes lightened at the end of fermentation. Similar to our results, the Mulyani et al. [46] study on water kefir found that the color of the product changed during fermentation.

**Figure 3:**
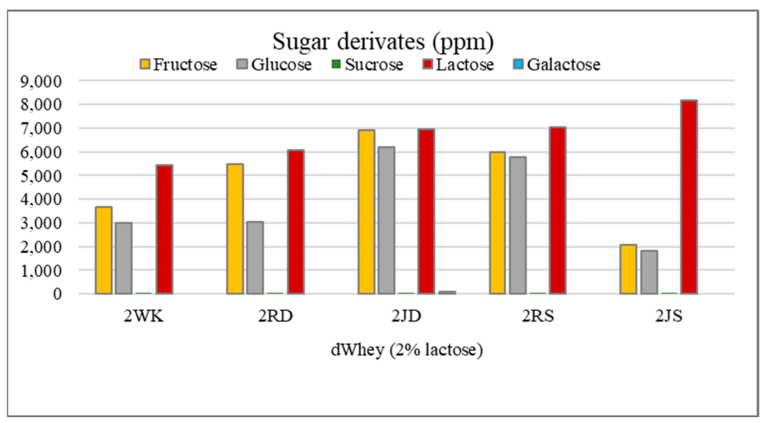
Sugar derivates of water kefir drinks produced from demineralized whey (dWhey) with 2% lactose content and with the addition of Dimrit and Shiraz raisins or grape juice. 2WK = 2% lactose; 2RD = 2% lactose + Dimrit raisin; 2JD = 2% lactose + Dimrit grape juice; 2RS = 2% lactose + Shiraz raisin; 2JS = 2% lactose + Shiraz grape juice.

**Figure 4:**
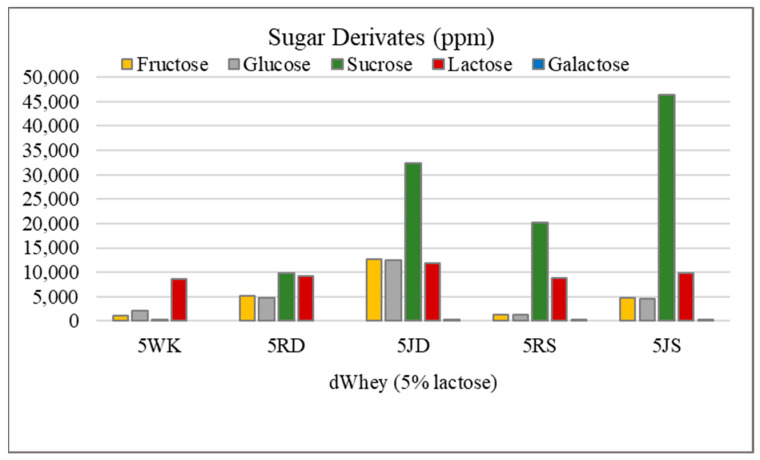
Sugar derivates of water kefir drinks produced from demineralized whey (dWhey) with 5% lactose content and with the addition of Dimrit and Shiraz raisins or grape juice. 5WK = 5% lactose; 5RD = 5% lactose + Dimrit raisin; 5JD = 5% lactose + Dimrit grape juice; 5RS = 5% lactose + Shiraz raisin; 5JS = 5% lactose + Shiraz grape juice.

The lactose content, the addition of grape raisins/juice, as well as the grape variety affected the sugar derivatives content in water kefir (Figure 3 and Figure 4). In particular, the amounts of sucrose, fructose, glucose and lactose were higher in the samples with 5% lactose in comparison to the samples with 2% lactose. Glucose, together with fructose, is the most important sugar derivative in fruit [47] and is popularly known as “grape sugar”. Sample 5JD had a significantly higher amount of fructose and glucose (*p* < 0.05), while the lowest amounts were found in samples 5WK and 5RS. There is significant difference (*p* < 0.05) between samples to which raisins were added compared to those with grape juice. The glucose content of the 2JD sample was about twice that of 2RD (*p* < 0.05).

However, the opposite was observed in the Shiraz grape and it was found that the glucose content of the 2RS sample was about three times higher than that of the 2JS (*p* < 0.05). The glucose content in the water kefir with Dimrit grape juice (5JD) was significantly higher (*p* < 0.05) compared to the water kefir with raisins (5JR). The sucrose content was higher in 5% lactose water kefir. The effects of adding raisins and grape juice as well as grape variety showed a statistically significant difference (*p* < 0.05). For both grape varieties addition of grape juice in the production of water kefir (5JD, 5JS) resulted in significantly higher (*p* < 0.05) sucrose content. The lactose content was lower in control samples, suggesting that the microflora present in water kefir tend to use other sugar derivates as a carbohydrate source when raisins or juice were added. In the absence of glucose, lactose is probably used as a carbohydrate source. The amount of galactose in the samples was determined at a low level. The fact that galactose was not detected in samples 2WK, 2RD, 2RS, 2JS, 5WK and 5RD suggests that it may be present in low concentration in the drink and converted to glucose by the microflora of the water kefir during fermentation. Sucrose is usually the main substrate during water kefir fermentation and is degraded by the microorganisms to ethanol, glycerol, lactic acid, acetic acid, mannitol and various flavor compounds [48]. The degradation of sucrose to its monosaccharides, namely, glucose and fructose, is carried out by the yeast group in the water kefir grain. Stadie et al. [49] found that the amount of glucose and fructose increased within 48 h after fermentation. The researchers determined a glucose and fructose content of 1.08% and 1.72%, respectively, in water kefir drinks containing 10% carrot juice. Similar results were obtained in water kefir drinks containing black tea and it was found that the amount of glucose increased from 0.46% to 0.97% and the amount of fructose increased from 0.34% to 1.58% as a result of the 72 h fermentation [50]. The presence of more fructose than glucose in the water kefir drink suggests that glucose is consumed faster after sucrose is broken down into its monosaccharides [51]. However, looking at the data obtained in our study, it was determined that the glucose and fructose contents of the samples were mostly similar.

Ethanol, as well as hexanoic, pentanoic and acetic acid were detected in the highest amount in most of the water kefir drinks. However, other volatile compounds such as 1-butanol 3-methyl acetate, ethyl acetate, 2-propanoic acid and butylated hydroxytoluene were also detected in some samples in varying amounts (Table 5). The lowest content of ethanol was detected in water kefir beverages produced with Shiraz raisins/juice. On the other hand, pentanoic acid was detected at the highest level in control samples (2WK and 5WK).

Laureys and De Vuyst [52] found that in addition to ethanol, acetaldehyde and acetic acid acetate are also formed during fermentation. These results indicate that yeast and acetic acid bacterial groups in the microflora of water kefir grains grow well. However, the acetic acid and ethanol content should not be too high in order not to suppress the fruity taste of water kefir. The highest amount of ethyl acetate was 1581.5 mg/kg in the control sample with 5% lactose content (5WK). In a study conducted by Laureys and De Vuyst [52], the ethyl acetate content was 13.4 mg/L. Patel et al. [53] state that the flavor of water kefir is mainly due to the high content of alcohols, aldehydes, acetic acid, lactic acid and gluconic acid. It is found that *Saccharomyces* in the microbiota of water kefir is responsible for the production of aldehydes and esters, while the non-Saccharomyces yeast group is responsible for the production of higher content of alcohols and esters. In a study with different fruit varieties, it was found that alcohols (2-hexenal, 1-hexanol and hexanal), aldehydes (nonanal) and esters (ethyl butanoate) have the strongest influence on water kefir flavor [54].

### 3.2. Microbiological Properties

The microbiota of water kefir is composed of lactic acid bacteria, acetic acid bacteria and yeasts, depending on the origin of the grain. However, as the acidity and carbohydrate content of the environment can change with the addition of fruits such as figs, apples and grapes, different dominant groups of the microbiota can develop [4,23].

**Figure 5:**
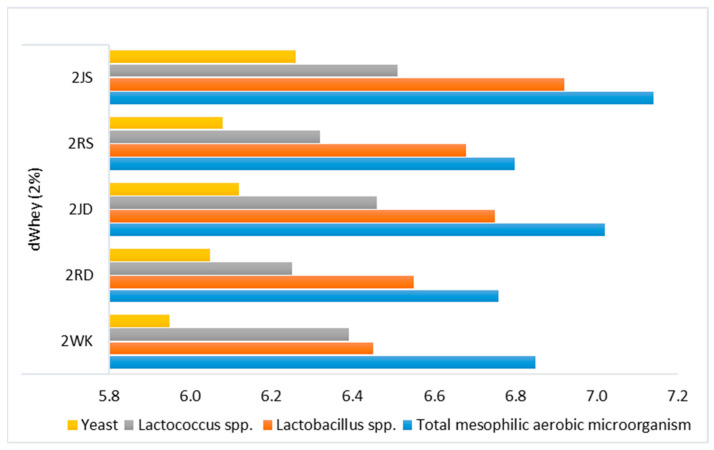
Microbiological characteristics (log cfu/mL) of water kefir drinks produced from demineralized whey (dWhey) with 2% lactose content and with the addition of Dimrit and Shiraz raisins or grape juice. 2WK = 2% lactose; 2RD = 2% lactose + Dimrit raisin; 2JD = 2% lactose + Dimrit grape juice; 2RS = 2% lactose + Shiraz raisin; 2JS = 2% lactose + Shiraz grape juice.

**Figure 6:**
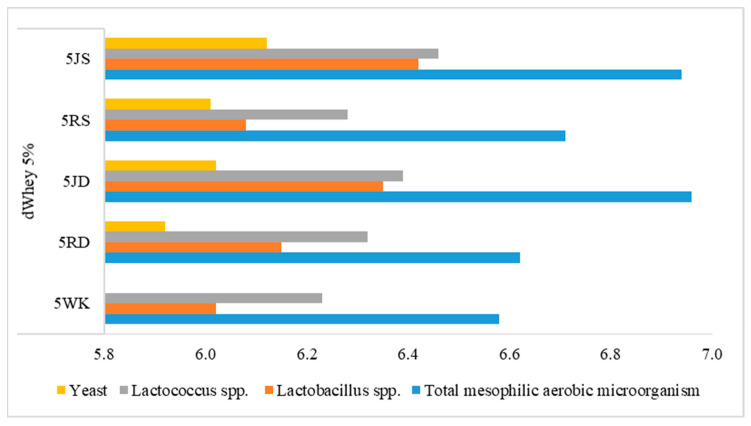
Microbiological characteristics (log cfu/mL) of water kefir drinks produced from demineralized whey (dWhey) with 5% lactose content and with the addition of Dimrit and Shiraz raisins or grape juice. 5WK = 5% lactose; 5RD = 5% lactose + Dimrit raisin; 5JD = 5% lactose + Dimrit grape juice; 5RS = 5% lactose + Shiraz raisin; 5JS = 5% lactose + Shiraz grape juice.

A noticeable number of mesophilic bacteria in control samples (log cfu/mL > 6) indicates that the lactose contained in the dWhey water kefir is utilized by the microorganisms and fermented to glucose and galactose, thus maintaining the viability of the microorganisms through glucose fermentation (Figure 5 and Figure 6). The highest number of mesophilic aerobic bacteria were detected in the samples containing grape juice, while the samples containing raisins were similar to the control sample. Therefore, sucrose, fructose and glucose from grape juice are considered to better meet the microorganisms’ needs for carbon sources during fermentation.

The number of *Lactobacillus* spp. was slightly higher in 2WK than in 5WK. In the samples containing raisins and grape juice, the number of *Lactobacillus* was lower in the samples with 5% lactose. The number of *Lactococcus* spp. was higher in the water kefir drinks with 5% lactose and grape juice than in those with raisins. Although the yeast numbers were similar in samples with both lactose concentrations, the influence of the carbohydrate sources from grape juice seems to be noticeable on the viability of the yeast groups. Therefore, it can be said that the number of microorganisms tends to decrease due to the influence of high lactose content on the acidity level during water kefir fermentation. Nevertheless, the substrates used in the production of water kefir, parameters such as composition, fermentation temperature and duration can influence the amount of metabolites at different levels [55]. In addition, cell enzymes and postbiotics after fermentation in probiotic drinks are also very important for human health [56].

### 3.3. Sensory Properties

**Figure 7:**
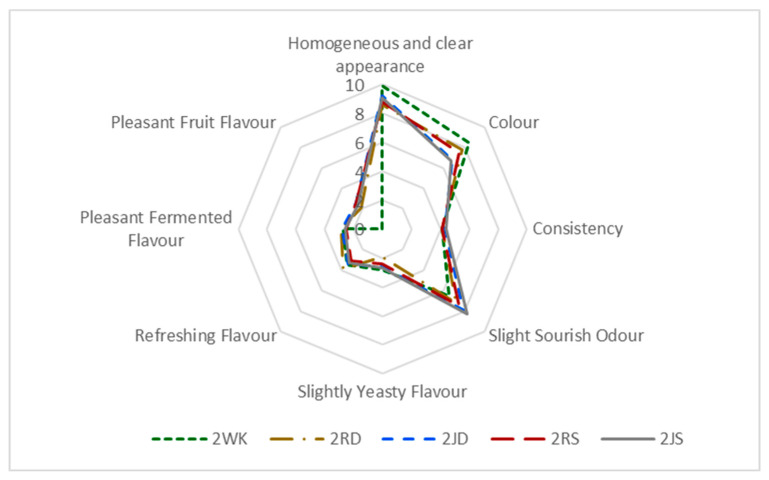
Sensorial evaluation of water kefir drinks produced from demineralized whey (dWhey) with 2% lactose content and with the addition of Dimrit and Shiraz raisins or grape juice. 2WK = 2% lactose; 2RD = 2% lactose + Dimrit raisin; 2JD = 2% lactose + Dimrit grape juice; 2RS = 2% lactose + Shiraz raisin; 2JS = 2% lactose + Shiraz grape juice.

**Figure 8:**
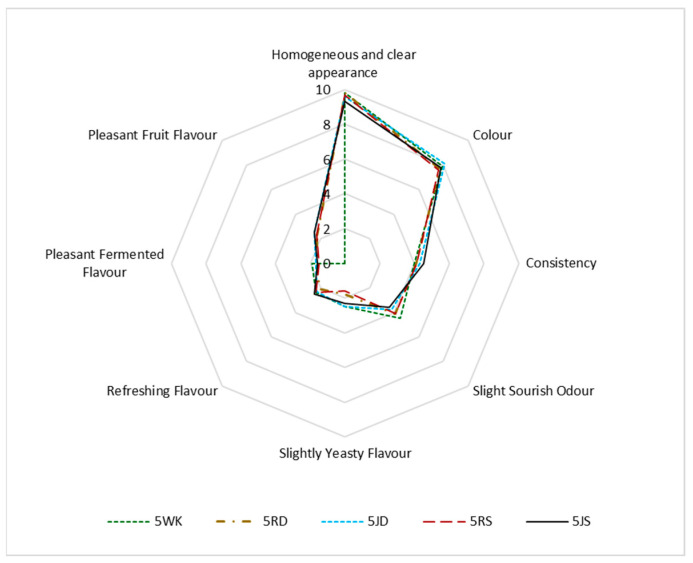
Sensorial evaluation of water kefir drinks produced from demineralized whey (dWhey) with 5% lactose content and with the addition of Dimrit and Shiraz raisins or grape juice. 5WK = 5% lactose; 5RD = 5% lactose + Dimrit raisin; 5JD = 5% lactose + Dimrit grape juice; 5RS = 5% lactose + Shiraz raisin; 5JS = 5% lactose + Shiraz grape juice.

Water kefir is not only a refreshing drink, but also stands out for its probiotic properties. The color of the drink can also vary depending on the sugar or fruit used (Figure 1). Since the color of dWhey is yellowish-green, at the end of fermentation it was observed that the color of the product, which was initially slightly yellowish-green and fruity, changed from a lighter yellowish-green shade to white in the control and raisin-containing samples and to light pink and light yellow in the grape juice-containing products. In terms of consistency in the mouth, it was found that the density perception of the drinks was slightly better in samples with added fruit, especially in drinks with grape juice. Usually, the biggest problem with whey drinks is the salty taste and cheesy odor [12]. Such a strong taste and odor was not detected in the products obtained in this study, which can be attributed to the use of diluted dWhey as a raw material (Figure 7 and Figure 8).

Lowering the pH of dWhey-based water kefirs containing 2% and 5% lactose was noticeable in the taste of the product and resulted in a more acidic taste and odor, especially at the 5% lactose concentration of the samples to which Shiraz grape juice was added (5JS). Oxidation of color was observed in all products, and the yellowish and pinkish color of the samples caused by the addition of Dimrit and Shiraz grapes changed to a lighter color after fermentation, resulting in the lower color ratings. Samples with higher lactose content were rated lower due to their sour, fermented and yeasty taste. The refreshing flavor of the water kefir with added raisins was similar to that of the control samples (Figure 7 and Figure 8).

It was found that the sensory quality of the two-day water kefir obtained from the whey of tofu cheese made from soy milk was better and had a brighter and clearer color than the soy whey [57]. The researchers explained that this could be due to the change in phenolic compounds. However, it was observed that a sharp and intense sour taste of the water kefir drink as a result of prolonged fermentation is not generally accepted and sensory ratings are low. In this study, the pleasant fermented flavor, as well as the refreshing flavor during the two-day fermentation period in water kefir production is due to the aromatic volatile compounds formed during this time (Table 5). The values for the refreshing flavor and the pleasant fermented flavor were higher for water kefir drinks with 2% lactose.

## 4. Conclusions

According to the results, 48 h fermentation of dWhey is promising in the production of water kefir drinks. Moreover, the addition of grape varieties combined with limited fermentation time (12 h) increased the total phenolic content and the total solids content. It can be concluded that the Dimrit grape, a local table grape variety of the Burdur province, can be used commercially for the production of dWhey-based water kefir drinks, as well as Shiraz grape. The Shiraz grape, grown as a wine grape variety in Uşak province, is suitable for the production of water kefir drink with numerous nutritional benefits, especially in terms of chemical composition, total phenolic content, antioxidant activity and microbiological properties. Finally, this study confirmed that it would be beneficial to produce water kefir drink from dWhey with 2% lactose to maintain better viability of kefir microbiota and obtain less acidic water kefir drink compared to 5% dWhey. When using dWhey and some grape varieties to produce water kefir drinks, future investigations could focus on the relationship between the duration of fermentation of dWhey alone, and the duration of fermentation after mixing dWhey with grape varieties. This could allow manipulation with different carbohydrate sources necessary for water kefir grain growth. In this way, physico-chemical, microbiological and sensory properties of water kefir drinks based on dWhey and specific grape varieties could be optimized.

## Figures and Tables

**Table 1:** Code of samples.

Code	Sample
2WK	Water kefir with 2% lactose (control group)
2RD	Water kefir drink with 2% lactose + Raisins of the Dimrit grape variety
2JD	Water kefir drink with 2% lactose + Grape juice of the Dimrit grape variety
2RS	Water kefir drink with 2% lactose + Raisins of the Shiraz grape variety
2JS	Water kefir drink with 2% lactose + Grape juice of the Shiraz grape variety
5WK	Water kefir with 5% lactose (control group)
5RD	Water kefir drink with 5% lactose + Raisins of the Dimrit grape variety
5JD	Water kefir drink with 5% lactose + Grape juice of the Dimrit grape variety
5RS	Water kefir drink with 5% lactose + Raisins of the Shiraz grape variety
5JS	Water kefir drink with 5% lactose + Grape juice of the Shiraz grape variety

**Table 2:** Chemical composition of water kefir drinks produced from demineralized whey (dWhey) with the addition of Dimrit and Shiraz raisins or grape juice.

Parameter	dWhey (2% Lactose)					dWhey (5% Lactose)				
	2WK	2RD	2JD	2RS	2JS	5WK	5RD	5JD	5RS	5JS
pH	3.71 ± 0.01 ^aA^	3.67 ± 0.00 ^bA^	3.66 ± 0.01 ^bA^	3.66 ± 0.01 ^bA^	3.56 ± 0.02 ^cA^	3.68 ± 0.01 ^aA^	3.61 ± 0.02 ^abA^	3.59 ± 0.02 ^bB^	3.62 ± 0.01 ^abA^	3.43 ± 0.02 ^cB^
Total Solid (%)	4.88 ± 0.01 ^cB^	5.12 ± 0.01 ^bA^	5.22 ± 0.02 ^aA^	4.87 ± 0.01 ^cB^	5.18 ± 0.01 ^bB^	4.95 ± 0.01 ^cA^	5.18 ± 0.02 ^baA^	5.25 ± 0.01 ^aA^	5.18 ± 0.01 ^bA^	5.26 ± 0.02 ^aA^
Protein (%)	0.12 ± 0.03 ^dB^	0.16 ± 0.03 ^cB^	0.20 ± 0.03 ^bB^	0.19 ± 0.03 ^bB^	0.30 ± 0.03 ^aB^	0.19 ± 0.03 ^cA^	0.30 ± 0.03 ^bA^	0.38 ± 0.03 ^aA^	0.30 ± 0.03 ^bA^	0.41 ± 0.04 ^aA^
Ash (%)	0.43 ± 0.01 ^bA^	0.44 ± 0.02 ^aB^	0.46 ± 0.01 ^aB^	0.46 ± 0.01 ^aB^	0.48 ± 0.01 ^aB^	0.44 ± 0.01 ^bA^	0.50 ± 0.02 ^aA^	0.51 ± 0.01 ^aA^	0.51 ± 0.02 ^aA^	0.53 ± 0.01 ^aA^
TSS (°Bx)	2.11 ± 0.01 ^eB^	3.12 ± 0.02 ^cB^	3.23 ± 0.02 ^bB^	2.51 ± 0.01 ^dB^	4.02 ± 0.02 ^aB^	5.12 ± 0.02 ^dA^	6.12 ± 0.01 ^aA^	6.03 ± 0.01 ^bA^	5.08 ± 0.02 ^dA^	5.80 ± 0.01 ^cA^
Viscosity (cP)	10.32 ± 0.2 ^cB^	12.02 ± 0.3 ^bA^	14.40 ± 0.2 ^aA^	10.20 ± 0.3 ^cB^	12.40 ± 0.2 ^bB^	11.20 ± 0.4 ^dA^	11.60 ± 0.2 ^dB^	14.80 ± 0.3 ^aA^	12.40 ± 0.2 ^cA^	13.20 ± 0.1 ^bA^
a_w_	0.920 ± 0.01 ^aA^	0.919 ± 0.02 ^aA^	0.921 ± 0.01 ^aA^	0.920 ± 0.01 ^aA^	0.921 ± 0.02 ^aA^	0.912 ± 0.02 ^aB^	0.912 ± 0.01 ^aB^	0.913 ± 0.01 ^aB^	0.912 ± 0.01 ^aB^	0.914 ± 0.01 ^aB^
TPC	24.1 ± 0.5 ^dB^	26.2 ± 0.1 ^cB^	28.3 ± 0.3 ^abB^	27.1 ± 0.2 ^bB^	29.6 ± 0.3 ^aB^	27.6 ± 0.2 ^cA^	27.5 ± 0.3 ^cA^	31.2 ± 0.2 ^bA^	30.6 ± 0.5 ^bA^	33.1 ± 0.2 ^aA^
DPPH	10.4 ± 0.6 ^eA^	13.4 ± 0.3 ^dA^	15.62 ± 0.5 ^bA^	14.2 ± 0.2 ^cA^	16.3 ± 0.1 ^aB^	11.2 ± 0.2 ^dA^	13.8 ± 0.3 ^cA^	14.9 ± 0.2 ^bA^	14.5 ± 0.1 ^bA^	17.3 ± 0.3 ^aA^
TEAC	12.44 ± 1.3 ^dB^	16.2 ± 0.7 ^cB^	18.6 ± 0.5 ^bA^	18.3 ± 0.8 ^bB^	24.8 ± 1.1 ^aA^	13.2 ± 0.5 ^aA^	17.3 ± 0.7 ^cA^	19.3 ± 0.5 ^bA^	21.2 ± 1.3 ^bA^	23.5 ± 0.4 ^aB^

2WK = 2% lactose; 2RD = 2% lactose + raisin of Dimrit; 2JD = 2% lactose + grape juice of Dimrit; 2RS = 2% lactose + raisin of Shiraz; 2JS = 2% lactose + grape juice of Shiraz; 5WK = 5% lactose; 5RD = 5% lactose + raisin of Dimrit; 5JD = 5% lactose + grape juice of Dimrit; 5RS = 5% lactose + raisin of Shiraz; 5JS = 5% lactose + grape juice of Shiraz. a_w_ = water activity; TSS = total soluble solids amount (°Bx); TPC = total phenolic content (mg GAE/L); DPPH antioxidant activity = 2,2,-diphenyl-2-picryl-hydrazyl (% İnhibition); TEAC antioxidant activity = Trolox equivalent antioxidant capacity (µM TE/100 g). Means within the same row marked with different uppercase letters differ significantly (*p* < 0.05) between dWhey samples of the same grape variety added, while lowercase letters showed significant differences (*p* < 0.05) within dWhey samples.

**Table 3:** Mineral content (µg/g) of water kefir drinks produced from demineralized whey (dWhey) with the addition of Dimrit and Shiraz raisins or grape juice.

Mineral	dWhey (2% Lactose)					dWhey (5% Lactose)				
	2WK	2RD	2JD	2RS	2JS	5WK	5RD	5JD	5RS	5JS
Zn	16.47 ± 0.21 ^aA^	14.86 ± 0.42 ^aA^	8.33 ± 0.08 ^bB^	9.94 ± 0.03 ^bB^	6.22 ± 0.61 ^cB^	13.69 ± 0.47 ^aB^	10.69 ± 0.27 ^bB^	12.68 ± 0.61 ^abA^	12.50 ± 0.21 ^abA^	10.18 ± 0.11 ^bA^
P	75.35 ± 2.13 ^cB^	192.21 ± 3.51 ^aA^	76.19 ± 0.67 ^cB^	161.23 ± 3.21 ^bA^	51.25 ± 0.49 ^dB^	121.35 ± 1.21 ^bA^	121.74 ± 6.21 ^bB^	93.78 ± 3.27 ^cA^	154.09 ± 6.15 ^aB^	86.48 ± 2.60 ^cA^
Mn	0.05 ± 0.03 ^cA^	0.191 ± 0.04 ^bB^	0.05 ± 0.03 ^cA^	0.243 ± 0.02 ^aA^	0.02 ± 0.01 ^cB^	0.05 ± 0.01 ^dA^	1.36 ± 0.03 ^aA^	0.09 ± 0.02 ^cA^	0.06 ± 0.03 ^cdB^	0.25 ± 0.04 ^bA^
Fe	6.80 ± 0.06 ^dA^	40.02 ± 0.16 ^aB^	11.13 ± 0.05 ^cB^	21.37 ± 0.63 ^bA^	13.71 ± 0.27 ^cA^	6.38 ± 0.19 ^cA^	82.35 ± 0.41 ^aA^	19.78 ± 1.13 ^bA^	18.80 ± 2.06 ^bA^	15.60 ± 3.31 ^bA^
Mg	35.46 ± 3.56 ^cB^	105.64 ± 3.41 ^aA^	40.11 ± 2.62 ^cA^	86.91 ± 1.33 ^bA^	27.19 ± 0.68 ^dA^	66.15 ± 2.33 ^aA^	15.34 ± 1.63 ^cB^	7.78 ± 0.41 ^dB^	20.35 ± 0.52 ^bB^	9.33 ± 0.73 ^dB^
Ca	158.9 ± 4.27 ^bB^	108.33 ± 3.12 ^dA^	168.52 ± 2.45 ^bA^	280.83 ± 6.54 ^aA^	121.10 ± 2.86 ^cB^	201.35 ± 4.91 ^aA^	38.50 ± 1.45 ^dB^	47.86 ± 2.78 ^dB^	112.21 ± 3.14 ^cB^	170.95 ± 2.14 ^bA^
Cu	26.64 ± 0.32 ^aA^	18.19 ± 0.57 ^bA^	18.61 ± 0.62 ^bA^	23.62 ± 0.36 ^aA^	21.86 ± 2.49 ^aA^	14.15 ± 0.47 ^aB^	6.20 ± 0.08 ^dB^	10.48 ± 0.38 ^cB^	11.85 ± 0.73 ^bcB^	12.14 ± 0.17 ^abB^
Na	92.50 ± 1.45 ^cB^	309.02 ± 3.62 ^aA^	96.29 ± 0.95 ^cB^	257.11 ± 3.41 ^bA^	55.40 ± 0.62 ^dA^	190.90 ± 2.35 ^aA^	125.25 ± 6.63 ^cB^	175.82 ± 3.13 ^bA^	20.50 ± 0.65 ^eB^	61.35 ± 0.39 ^dA^
K	207.85 ± 6.28 ^cA^	450.34 ± 5.31 ^aA^	166.63 ± 1.85 ^dB^	333.87 ± 7.12 ^bA^	107.40 ± 6.24 ^eB^	241.25 ± 5.43 ^bA^	260.85 ± 7.84 ^bB^	234.66 ± 4.29 ^bA^	330.26 ± 9.45 ^aA^	351.70 ± 7.03 ^aA^

2WK = 2% lactose; 2RD = 2% lactose + raisin of Dimrit; 2JD = 2% lactose + grape juice of Dimrit; 2RS = 2% lactose + raisin of Shiraz; 2JS = 2% lactose + grape juice of Shiraz; 5WK = 5% lactose; 5RD = 5% lactose + raisin of Dimrit; 5JD = 5% lactose + grape juice of Dimrit; 5RS = 5% lactose + raisin of Shiraz; 5JS = 5% lactose + grape juice of Shiraz. Means within the same row marked with different uppercase letters differ significantly (*p* < 0.05) between dWhey samples of the same grape variety added, while lowercase letters showed significant differences (*p* < 0.05) within dWhey samples.

**Table 4:** Color value (CIE L*, a*, b*) of water kefir produced from demineralized whey (dWhey) with the addition of Dimrit and Shiraz raisins or grape juice.

Parameter	dWhey (2% Lactose)					dWhey (5% Lactose)				
	2WK	2RD	2JD	2RS	2JS	5WK	5RD	5JD	5RS	5JS
L*	21.99 ± 1.23 ^eA^	22.27 ± 0.82 ^cA^	22.69 ± 1.21 ^aA^	22.50 ± 0.16 ^bA^	22.17 ± 0.45 ^dA^	21.87 ± 0.52 ^bA^	21.68 ± 0.42 ^cB^	22.67 ± 0.31 ^aA^	22.06 ± 0.28 ^bB^	21.62 ± 0.19 ^cB^
a*	0.15 ± 0.03 ^bA^	−0.11 ± 0.02 ^eA^	−0.07 ± 0.01 ^dA^	−0.03 ± 0.03 ^cA^	0.53 ± 0.02 ^aA^	−0.08 ± 0.01 ^bB^	−0.32 ± 0.02 ^dB^	−0.53 ± 0.06 ^eB^	−0.23 ± 0.05 ^cB^	0.04 ± 0.01 ^aB^
b*	0.08 ± 0.04 ^cA^	0.64 ± 0.02 ^bA^	1.31 ± 0.03 ^aA^	0.10 ± 0.05 ^cA^	0.59 ± 0.08 ^bA^	0.13 ± 0.03 ^dA^	0.53 ± 0.07 ^aB^	0.35 ± 0.06 ^cB^	0.03 ± 0.01 ^eB^	0.48 ± 0.09 ^bB^

2WK = 2% lactose; 2RD = 2% lactose + raisin of Dimrit; 2JD = 2% lactose + grape juice of Dimrit; 2RS = 2% lactose + raisin of Shiraz; 2JS = 2% lactose + grape juice of Shiraz; 5WK = 5% lactose; 5RD = 5% lactose + raisin of Dimrit; 5JD = 5% lactose + grape juice of Dimrit; 5RS = 5% lactose + raisin of Shiraz; 5JS = 5% lactose + grape juice of Shiraz. Means within the same row marked with different uppercase letters differ significantly (*p* < 0.05) between dWhey samples of the same grape variety added, while lowercase letters showed significant differences (*p* < 0.05) within dWhey samples.

**Table 5:** Volatile components content (mg/kg) of water kefir drinks produced from demineralized whey (dWhey) with the addition of Dimrit and Shiraz raisins or grape juice.

		dWhey (2% Lactose)					dWhey (5% Lactose)				
RT	Volatile Compounds	2WK	2RD	2JD	2RS	2JS	5WK	5RD	5JD	5RS	5JS
3.860	Carbon dioxide	6.39	n.d.	n.d.	n.d.	n.d.	n.d.	n.d.	n.d.	n.d.	n.d.
4.081	Ethyl Hydrogene Oxalate	n.d.	n.d.	n.d.	n.d.	16.92	n.d.	n.d.	n.d.	n.d.	n.d.
4.434	n-Hyx Methylamine	n.d.	n.d.	n.d.	n.d.	2.77	n.d.	n.d.	n.d.	n.d.	n.d.
5.836	Acetaldehyde	n.d.	n.d.	2.39	n.d.	4.64	50.35	n.d.	3.76	n.d.	n.d.
5.997	Ethyl Acetate	n.d.	21.74	n.d.	n.d.	69.2	1581.5	n.d.	35.6	n.d.	n.d.
6.732	Ethanol	147.23	298.11	77.06	142.5	6519.6	127.3	3.02	45.6	1.45	41.34
7.322	Formic Acid	n.d.	n.d.	n.d.	n.d.	n.d.	n.d.	12.2	n.d.	39.4	n.d.
7.828	Diisoamyl Oxalate	n.d.	n.d.	n.d.	0.09	n.d.	n.d.	n.d.	n.d.	n.d.	n.d.
8.119	Oxalic Acid	n.d.	n.d.	n.d.	7.04	n.d.	n.d.	74.6	178.6	288.2	115.19
8.349	Isoprophyl 5-Methyl Hexen-2-On	n.d.	n.d.	n.d.	n.d.	n.d.	n.d.	n.d.	5.82	n.d.	n.d.
9.045	Butanoic Acid 2-Methyl	n.d.	n.d.	169.8	n.d.	103.3	9140.4	n.d.	n.d.	n.d.	n.d.
9.373	Ethyl Methyl Acethylchloride	n.d.	0.77	n.d.	n.d.	n.d.	n.d.	n.d.	n.d.	n.d.	n.d.
9.536	Disulfide Dimethyl	n.d.	3.33	n.d.	1.03	n.d.	59.9	n.d.	n.d.	n.d.	n.d.
9.546	2-Furan Metanol	n.d.	n.d.	n.d.	n.d.	n.d.	n.d.	n.d.	n.d.	n.d.	n.d.
10.08	1-Propanol 2-Methyl	n.d.	15.9	3.95	2.52	n.d.	713.9	n.d.	n.d.	n.d.	n.d.
10.26	4-Heptanone 2-Methyl	n.d.	n.d.	n.d.	n.d.	n.d.	n.d.	2.28	n.d.	1.7	n.d.
10.61	1-Butanol- 3 Methyl	n.d.	n.d.	n.d.	5.45	n.d.	n.d.	n.d.	n.d.	n.d.	n.d.
10.64	1-Butanol 3-Methil Acetate	n.d.	n.d.	6.61	n.d.	9.36	n.d.	0.33	3.69	n.d.	n.d.
10.66	1-Butanol 2-Methyl	57.03	6.76	n.d.	n.d.	9.49	363.3	n.d.	n.d.	0.32	7.14
10.67	Butanoic Acid 2-Methyl	n.d.	513.7	n.d.	3.86	n.d.	n.d.	n.d.	n.d.	n.d.	n.d.
10.69	Toluen	n.d.	50.75	n.d.	n.d.	n.d.	n.d.	n.d.	n.d.	n.d.	n.d.
12.43	2-Heptanone	n.d.	2.69	n.d.	2.78	n.d.	138.1	n.d.	n.d.	n.d.	n.d.
13.12	1-Heptanol	n.d.	40.11	4.49	93.86	353.5	n.d.	n.d.	n.d.	n.d.	n.d.
13.96	3-Octanone	n.d.	n.d.	n.d.	n.d.	n.d.	n.d.	n.d.	n.d.	n.d.	0.72
13.98	Hexanoic Acid	n.d.	1302.6	950	123.4	8787.1	93,112.1	33.75	1.21	n.d.	n.d.
14.96	4-Octanone	n.d.	n.d.	n.d.	n.d.	n.d.	n.d.	n.d.	0.6	1.92	n.d.
15.89	2-Butanone	n.d.	n.d.	n.d.	n.d.	n.d.	n.d.	n.d.	0.24	n.d.	n.d.
15.90	2-Prophenol 1-Metoxy	n.d.	6.18	n.d.	n.d.	n.d.	n.d.	n.d.	n.d.	n.d.	n.d.
16.40	2-Propanoic Acid	n.d.	715.8	14.63	62.8	2.14	n.d.	1237.5	n.d.	n.d.	32.07
16.45	2-Propionic Acid	256.4	n.d.	n.d.	11.78	n.d.	n.d.	n.d.	46.75	n.d.	n.d.
16.45	Pentanoic Acid	11,040	12.66	630.1	2.1	783.4	12,765.5	25.43	n.d.	n.d.	n.d.
16.90	Methoxyacetic Acid	n.d.	n.d.	n.d.	n.d.	n.d.	n.d.	n.d.	n.d.	n.d.	10.77
17.63	Heptanoic Acid	n.d.	843.7	n.d.	4.34	1239.1	n.d.	n.d.	11.64	n.d.	n.d.
17.77	2-Pentene	n.d.	n.d.	n.d.	n.d.	n.d.	n.d.	1.38	n.d.	0.34	n.d.
21.96	Octanoic Acid	1019.7	n.d.	7017.2	216.7	n.d.	n.d.	125.7	n.d.	n.d.	20.21
22.91	Acetic Acid	n.d.	623.9	231.6	63.4	n.d.	8402.9	84.37	n.d.	131.9	137.15
23.38	7-Octene-2-Ol	n.d.	n.d.	n.d.	n.d.	n.d.	n.d.	n.d.	n.d.	n.d.	0.29
24.39	1-Hexanol 2- Ethyl	n.d.	52.62	n.d.	4.76	17.78	n.d.	2.05	0.97	1.54	3.26
26.23	Benzaldehyde	n.d.	23.45	1.58	n.d.	n.d.	n.d.	n.d.	n.d.	n.d.	n.d.
26.78	Nonanoic acid	270.03	1725.2	136	n.d.	n.d.	n.d.	26.65	n.d.	n.d.	n.d.
28.28	2-Heptanol	n.d.	n.d.	n.d.	n.d.	n.d.	60.9	n.d.	n.d.	n.d.	n.d.
29.84	1-Octanal	17.44	n.d.	n.d.	n.d.	4.23	n.d.	n.d.	n.d.	n.d.	0.2
30.67	1-Hexene	n.d.	n.d.	n.d.	2.11	n.d.	n.d.	n.d.	n.d.	n.d.	n.d.
31.60	1-Pentene	n.d.	n.d.	n.d.	n.d.	1.08	5.02	n.d.	0.72	0.34	0.26
31.42	6-Methy l-Octanal	n.d.	n.d.	n.d.	n.d.	n.d.	n.d.	0.98	n.d.	0.93	n.d.
33.57	1-Nonanol	n.d.	n.d.	n.d.	3.36	n.d.	n.d.	n.d.	0.33	0.45	0.15
33.76	Butanoic Acid	n.d.	201.6	n.d.	n.d.	n.d.	n.d.	n.d.	n.d.	n.d.	n.d.
35.17	Ethyl 9-Decenoate	n.d.	n.d.	1.04	n.d.	n.d.	n.d.	n.d.	n.d.	n.d.	n.d.
37.00	2-Nonane	n.d.	n.d.	n.d.	0.38	0.78	21.6	n.d.	n.d.	0.42	n.d.
38.47	Benzoic Acid	n.d.	673.4	51.58	1.74	n.d.	n.d.	n.d.	n.d.	n.d.	n.d.
45.85	Butylated Hydroxytoluen	7.99	n.d.	7.05	3.73	n.d.	n.d.	1.06	0.35	1.07	0.37
46.39	1-4-Butanediol	n.d.	2.09	n.d.	n.d.	n.d.	n.d.	n.d.	n.d.	n.d.	n.d.
73.58	Dibuthylphytalate	n.d.	n.d.	n.d.	0.27	n.d.	n.d.	n.d.	n.d.	n.d.	n.d.
87.39	n-Hexadecanoic Acid	n.d.	n.d.	n.d.	n.d.	2.46	n.d.	n.d.	n.d.	n.d.	n.d.
88.64	2-Hexanal	n.d.	n.d.	n.d.	n.d.	0.87	n.d.	n.d.	n.d.	n.d.	n.d.
88.76	Propene	n.d.	n.d.	n.d.	n.d.	n.d.	0.45	n.d.	n.d.	0.99	0.49
93.23	Squalene	n.d.	n.d.	n.d.	n.d.	n.d.	14.18	n.d.	n.d.	n.d.	n.d.

2WK = 2% lactose; 2RD = 2% lactose + raisin of Dimrit; 2JD = 2% lactose + grape juice of Dimrit; 2RS = 2% lactose + raisin of Shiraz; 2JS = 2% lactose + grape juice of Shiraz; 5WK = 5% lactose; 5RD = 5% lactose + raisin of Dimrit; 5JD = 5% lactose + grape juice of Dimrit; 5RS = 5% lactose + raisin of Shiraz; 5JS = 5% lactose + grape juice of Shiraz; RT: retention time; n.d.: not detected.

## Data Availability

The data are available from the corresponding author.
